# Impact of Tricuspid Regurgitation Severity and Right Ventricular Function on the Correlation and Agreement Between Transthoracic Echocardiography- and Right Heart Catheterization-Derived Systolic Pulmonary Artery Pressure

**DOI:** 10.7759/cureus.99318

**Published:** 2025-12-15

**Authors:** Taki Eddine Sid, Verena Bauer, Christian Stumpf

**Affiliations:** 1 Cardiology, Klinikum Bayreuth, Medizincampus Oberfranken-Friedrich-Alexander-Universität Erlangen-Nürnberg (FAU), Bayreuth, DEU

**Keywords:** pulmonary hypertension, right heart catheterization, right ventricular function, systolic pulmonary artery pressure, transthoracic echocardiography, tricuspid regurgitation

## Abstract

Background

Accurate estimation of systolic pulmonary artery pressure (SPAP) is essential for evaluating pulmonary hypertension (PH). Transthoracic echocardiography (TTE) provides a noninvasive alternative to right heart catheterization (RHC). This study aimed to assess the correlation and agreement between TTE- and RHC-derived SPAP and to evaluate the influence of tricuspid regurgitation (TR) severity, right ventricular (RV) function, and PH subtype on the reliability of TTE-derived measurements.

Methods

A retrospective analysis was conducted on patients who underwent TTE and RHC within a seven-day interval. Patient demographics, as well as TTE and RHC parameters, were represented using the median and interquartile range (IQR). Correlation and Bland-Altman analyses were performed, with subgroup evaluations based on RV function, TR severity, and PH subtype (pre-, post-, and combined PH). Regression analysis was used to assess the impact of RV function (measured by tricuspid annular plane systolic excursion (TAPSE)) and TR (assessed by vena contracta (VC)) on the difference between TTE and RHC (ΔSPAP) measurements.

Results

A total of 112 patients were included. The median SPAP was 47 mmHg by TTE compared to 44.5 mmHg by RHC (p = 0.36). Bland-Altman analysis revealed wide limits of agreement (-27.6 to +30.0 mmHg), with 46% (n = 52) of measurements differing by >10 mmHg. Linear regression analysis did not reveal any significant effects of TAPSE and VC on ΔSPAP (R² = 0.018, p = 0.496). The overall correlation between TTE-SPAP and RHC-SPAP was moderate (ρ = 0.61, p < 0.001). In subgroup analysis, a stronger correlation was observed in preserved RV function (ρ = 0.78 vs. 0.48 in impaired RV function). Moderate correlation was found in both precapillary (ρ = 0.68, p < 0.01) and combined PH (ρ = 0.66, p < 0.001), while postcapillary PH showed nonsignificant correlation (ρ = 0.37, p = 0.197).

Conclusion

TTE provides a useful noninvasive estimate of SPAP but remains limited in terms of accuracy and consistency in specific clinical settings. The lack of predictive value of RV function and TR severity suggests that other physiological factors underlie the observed discordance with RHC. Therefore, RHC should remain the reference standard for precise assessment of pulmonary artery pressure.

## Introduction

Transthoracic echocardiography (TTE) is the primary noninvasive modality for assessing systolic pulmonary arterial pressure (SPAP) in the diagnosis and management of pulmonary hypertension (PH) [1]. It also has prognostic value in various cardiovascular and respiratory conditions, including heart failure, chronic obstructive pulmonary disease (COPD), and pulmonary embolism [2-8]. Furthermore, SPAP has become essential in the context of tricuspid regurgitation (TR), facilitating patient selection and planning for transcatheter interventions, including edge-to-edge repair (TEER) and valve replacement (TTVR) [9,10].

Despite its use, the agreement between TTE-derived and right heart catheterization (RHC)-derived SPAP measurements remains debatable. Previous studies have reported inconsistent results, with accuracy often reduced in severe TR [11-15]. Additionally, the influence of right ventricular (RV) systolic function on the discrepancy between TTE and RHC has not been comprehensively explored. Moreover, following the updated 2022 European Society of Cardiology (ESC)/European Respiratory Society (ERS) definition of PH (mean pulmonary artery pressure (mPAP) >20 mmHg; pulmonary vascular resistance (PVR) >2 Wood units) [1], limited data exist evaluating this relationship.

This study aimed to assess the correlation and agreement between TTE- and RHC-derived SPAP and to evaluate the effects of TR severity, RV function, and PH subtype (precapillary, postcapillary, and combined) on measurement variability.

An abstract based on this manuscript has been submitted to the ACC.26 (American College of Cardiology) Annual Scientific Session, to be held March 28-30, 2026, in New Orleans, LA.

## Materials and methods

Study population

This retrospective study analyzed electronic patient records from the Cardiology Department of Bayreuth Hospital, Bayreuth, Germany, over the past seven years. We included all patients who underwent both TTE and RHC during the same hospital admission between September 19, 2017, and January 21, 2025. The following exclusion criteria were applied: age <18 years; absence of tricuspid regurgitation; an interval exceeding seven days between TTE and RHC; inadequate assessment of echocardiographic or invasive hemodynamic measurement parameters (SPAP); and incomplete echocardiographic examination.

The study was approved by the Ethics Committee of the Friedrich-Alexander-Universität Erlangen-Nürnberg and conducted in accordance with the Declaration of Helsinki.

Doppler echocardiography

Doppler echocardiography was performed using EPIQ CVx 3D, EPIQ 7G, and CX50 ultrasound systems (Philips Healthcare, Andover, MA), equipped with phased-array transducers (X5-1; S5-1, 1-5 MHz). Estimation of SPAP comprised two principal components [7,8]: (1) continuous-wave Doppler measurement of the tricuspid regurgitant jet velocity, which was subsequently converted into a pressure gradient (between the right ventricle and right atrium) using the modified Bernoulli equation (ΔP = 4 × (TRV)²); (2) estimation of the right atrial pressure (RAP) based on the inferior vena cava (IVC) diameter and its collapse during inspiration. SPAP was then calculated as SPAP = 4 × (TRV)² + RAP.

In accordance with the American Society of Echocardiography (ASE) guidelines [8], RAP was categorized into three groups: if the IVC diameter is ≤21 mm with >50% collapse, RAP is 3 mmHg; if the IVC diameter is >21 mm with <50% collapse, RAP is 15 mmHg; and if only one criterion is met, RAP is 8 mmHg. SPAP was calculated as the sum of the right ventricle/right atrium pressure gradient and the RAP. The degree of tricuspid regurgitation was assessed using a semi-quantitative method, vena contracta (VC). Right ventricular function was assessed using tricuspid annular plane systolic excursion (TAPSE) [8,16].

All echocardiographic measurements were evaluated by an independent, experienced (>15-year experience) echocardiographer who was blinded to the RHC.

Right heart catheterization

RHC was conducted in the catheterization laboratory using hemodynamic monitoring systems from Siemens (Siemens Healthineers, Erlangen, Germany) and Philips (Philips Healthcare, Best, Netherlands). RHC was performed under local anesthesia via femoral or brachial access using the Seldinger technique, with hemodynamics obtained using a Swan-Ganz or multipurpose catheter under fluoroscopy and continuous monitoring. Systemic blood pressure was recorded invasively when combined with left heart catheterization or noninvasively using an oscillometric cuff.

The following hemodynamic parameters were recorded: mean pulmonary artery pressure (mPAP), SPAP, diastolic pulmonary artery pressure (DPAP), RAP, right ventricular systolic pressure (RVSP), pulmonary capillary wedge pressure (PCWP), and pulmonary vascular resistance (PVR). Cardiac output (CO) was measured using the Fick method.

Pulmonary hypertension (PH) was defined according to the current ESC/ERS guidelines [1]. Normal pulmonary artery pressure was defined as mPAP <21 mmHg. PH was diagnosed when mPAP was ≥21 mmHg. Postcapillary PH was defined as a PCWP >15 mmHg, precapillary PH as a PVR >160 dyn·s·cm⁻⁵ (>2 Wood units), and combined pre- and postcapillary PH when both PCWP and PVR were elevated. 

Statistical analysis

The normality of continuous variables was assessed using the Shapiro-Wilk test, which confirmed a nonnormal distribution. Continuous data are presented as medians with interquartile ranges (IQR) and categorical variables as frequencies and percentages. Comparisons were performed using the Wilcoxon signed-rank test. Agreement between TTE- and RHC-derived SPAP was evaluated using Bland-Altman analysis. ΔSPAP (TTE - RHC) was calculated, and linear regression was applied to examine the effects of TAPSE and vena contracta on ΔSPAP. Correlations were assessed using Spearman’s rank coefficient and stratified by TR severity, RV function, and PH subgroups. Statistical significance was set at p <0.05. Analyses were performed using JASP software (version 0.95; JASP Team, 2025).

## Results

**Figure 1:**
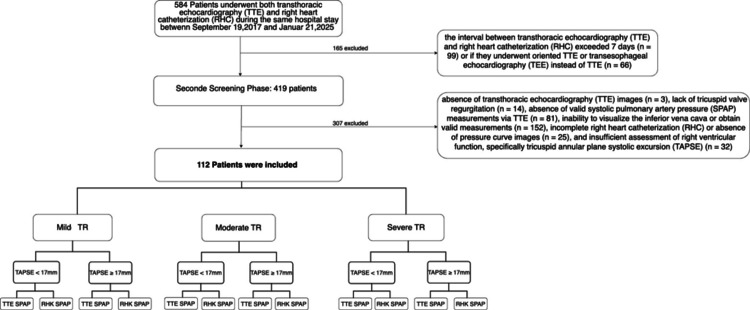
Patient Screening and Eligibility Flowchart A total of 584 patients were screened for inclusion. After exclusion of 470 patients based on predefined criteria, 112 patients met the eligibility requirements and were included in the final analysis.

From the initial cohort of 584 patients, 470 were excluded from the study, resulting in a final sample of 112 patients for analysis (Figure 1).

Notably, none of these patients experienced major cardiac events, underwent surgical interventions between TTE and RHC assessments or received targeted therapy for PH.

The median age was 77 years (IQR: 68-82), and 62 (54.4%) were female. The most frequent comorbidities were heart failure (n = 104 (92.9%)) and systemic arterial hypertension (n = 95 (83.3%)). Among those with heart failure, heart failure with preserved ejection fraction (HFpEF) was predominant and observed in 72 (69.2%) patients. A detailed summary of the baseline demographic and clinical characteristics is presented in Table 1.

**Table 1:** Baseline Demographic and Clinical Characteristics of the Study Cohort (N=112) Continuous variables are expressed as median (interquartile range, IQR), and categorical variables are presented as frequency and percentage (%). y: years; n: number; NYHA: New York Heart Association; CAD: coronary artery disease; PCI: percutaneous coronary intervention; CABG: coronary artery bypass grafting; COLD: chronic obstructive lung disease; CKD: chronic kidney disease; GFR: glomerular filtration rate; HLD: hyperlipidemia; DM: diabetes mellitus; ASD: atrial septal defect; PE: pulmonary embolism; DVT: deep vein thrombosis; BSA: body surface area; Hb: hemoglobin; NT-proBNP: N-terminal pro B-type natriuretic peptide

Variable	n (%) or Median (IQR)
Age	77 (68–82)
Sex	
F	62 (54.4)
M	50 (43.9)
CAD	58 (50.9)
PCI/CABG	30 (26.3)
Heart failure (NYHA)	104 (92.9)
I	8 (7.0)
II	28 (24.6)
III	61 (53.5)
IV	15 (13.4)
COLD	25 (21.9)
Pulmonary fibrosis	4 (3.5)
CKD (GFR mL/min)	58 (51.8)
Liver disease	6 (5.3)
HLD	42 (36.8)
DM	24 (21.1)
Hypertension	95 (83.3)
Atrial fibrillation	65 (57.0)
ASD	4 (3.5)
PE	4 (3.5)
Stroke/TIA	12 (10.5)
Smoking status	9 (7.9)
DVT	1 (0.9)
BSA – median	1.84 (1.71–1.99)
Hb (g/dL)	12.8 (11.5–14.3)
Creatinine (mg/dL)	1.1 (0.87–1.59)
Kalium (mmol/L)	4.2 (3.8–4.4)
Natrium (mmol/L)	139 (136–141)
NT-proBNP (pg/mL)	1894 (829–4888)

TTE and RHC were performed on the same day in 23 (20.5%) patients. Fifty-three (47.3%) underwent both procedures within one to two days, and 29 (18.7%) within three to five days. Longer intervals were less common (six days in five (4.5%) patients and seven days in two (1.8%) patients). The baseline hemodynamic and echocardiographic data are presented in Table 2.

**Table 2:** Baseline Echocardiographic and Hemodynamic Parameters of the Study Population, Including Both TTE and RHC Findings Continuous variables are expressed as median (interquartile range, IQR), while categorical variables are shown as frequency and percentage (%). TTE: transthoracic echocardiography; LVEF: left ventricular ejection fraction; TAPSE: tricuspid annular plane systolic excursion; SPAP: systolic pulmonary artery pressure; RA: right atrium; RV: right ventricle; TR: tricuspid regurgitation; MR: mitral regurgitation; MS: mitral stenosis; AR: aortic regurgitation; AS: aortic stenosis; RHC: right heart catheterization; RAPm: mean right atrial pressure; RVSP: right ventricular systolic pressure; DPAP: diastolic pulmonary artery pressure; MPAP: mean pulmonary artery pressure; PCWPm: mean pulmonary capillary wedge pressure; CO: cardiac output; CI: cardiac index; PVR: pulmonary vascular resistance

Parameter	Median (IQR) or Frequency (%)
Transthoracic Echocardiography	
LVEF (%)	55 (45–58.25)
TAPSE (mm)	19 (16–22)
SPAP (mmHg)	47 (35–60)
RAP (mmHg)	7 (8–15)
RV/RA Gradient (mmHg)	37.5 (27–50)
TAPSE/SPAP Ratio	0.41 (0.29–0.59)
Vena Contracta (mm)	6 (5–9)
TR Grade	
I	31 (27.7)
II	44 (39.3)
III	37 (33.0)
MR	105 (93.7)
MS	20 (17.9)
AR	57 (50.9)
AS	42 (37.5)
TAPSE (mm)	
<17 (Low)	45 (39.3)
≥17 (Normal)	69 (60.7)
Right heart Catheterization	
RAPm (mmHg)	7 (4–12)
RVSP (mmHg)	45.5 (34.75–56.25)
SPAP (mmHg)	44.5 (33.75–58)
DPAP (mmHg)	17 (12–23.5)
MPAP (mmHg)	27 (21–37)
PCWPm (mmHg)	18 (12–23)
CO (L/min)	3.85 (3.09–4.64)
CI (L/min/m²)	2.06 (1.70–2.46)
PVR (dyn·s·cm⁻⁵)	235.3 (132.1–370.8)
TAPSE/SPAP Ratio	0.40 (0.27–0.68)

**Figure 2:**
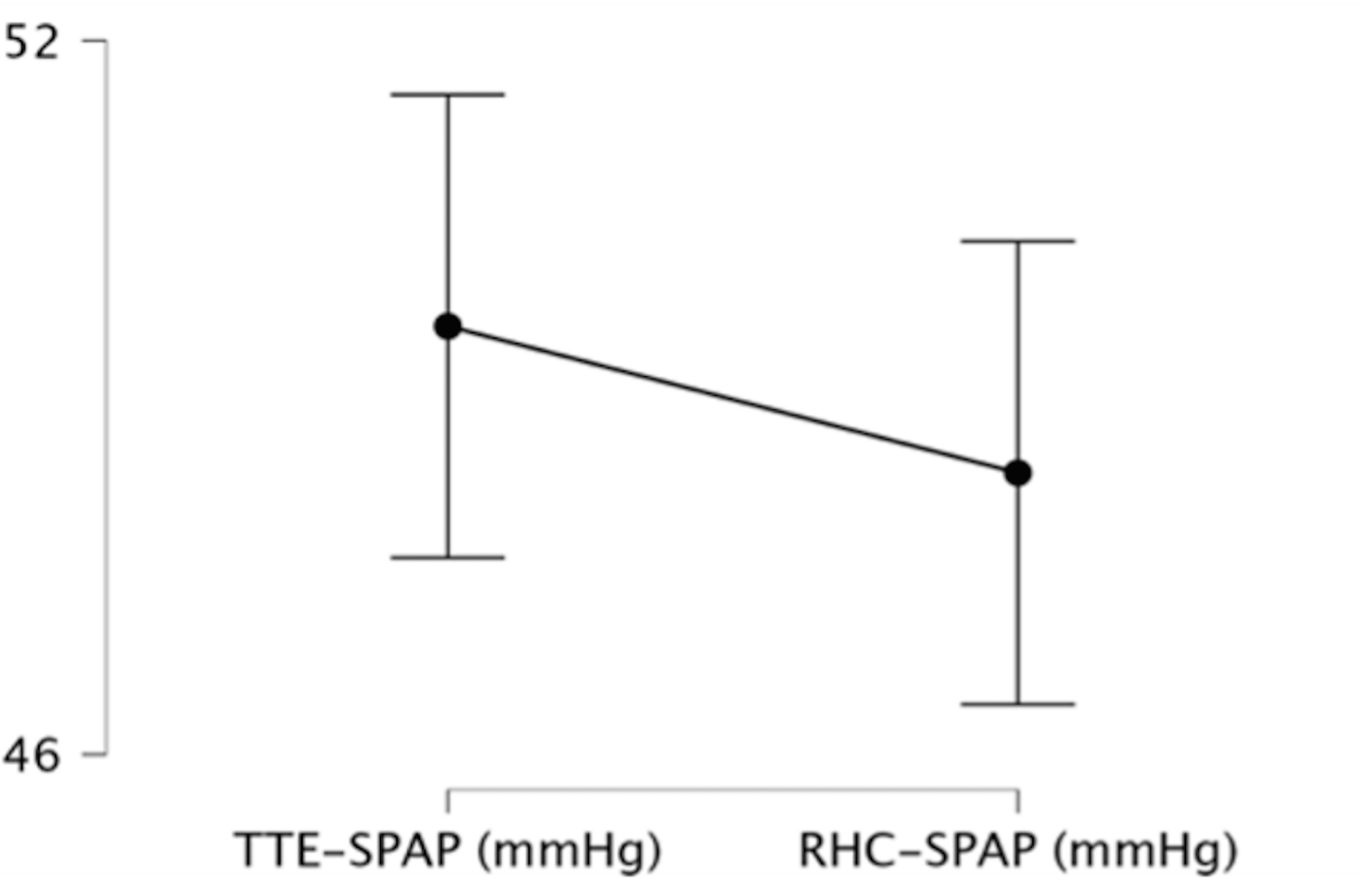
Comparison of Systolic Pulmonary Artery Pressure (SPAP) Measured by Transthoracic Echocardiography (TTE) and Right Heart Catheterization (RHC) Comparison of TTE- and RHC-derived SPAP values. No significant difference was observed (Wilcoxon signed-rank test; p=0.36).

The median SPAP was 47 mmHg (IQR: 35-60) by TTE and 44.5 mmHg (IQR: 34-58) by RHC, with no significant difference (p = 0.36; Figure 2).

**Figure 3:**
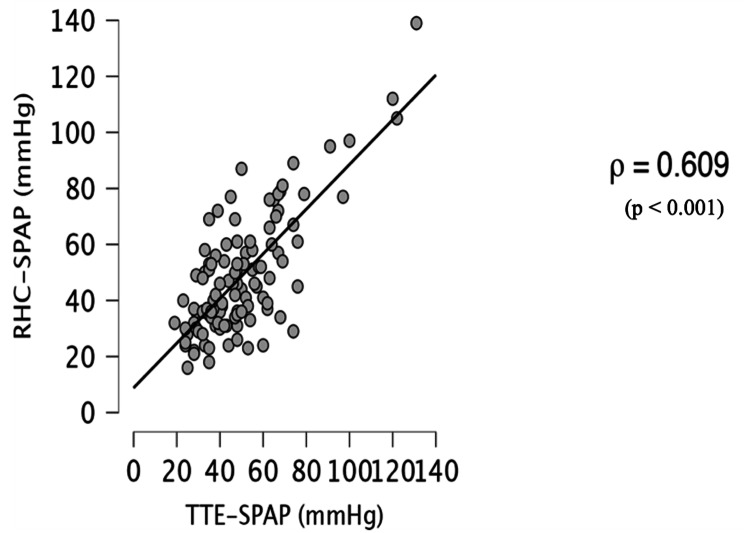
Correlation Between Systolic Pulmonary Artery Pressure (SPAP) Derived From Transthoracic Echocardiography (TTE) and Right Heart Catheterization (RHC) Scatter plot illustrating the correlation between SPAP estimated by TTE and that measured by RHC in patients with postcapillary pulmonary hypertension. A moderate, statistically significant positive correlation was observed (Spearman’s rank correlation coefficient: ρ = 0.609; p < 0.001).

The overall correlation was moderate (Spearman’s ρ = 0.609; p < 0.001; Figure 3).

**Figure 4:**
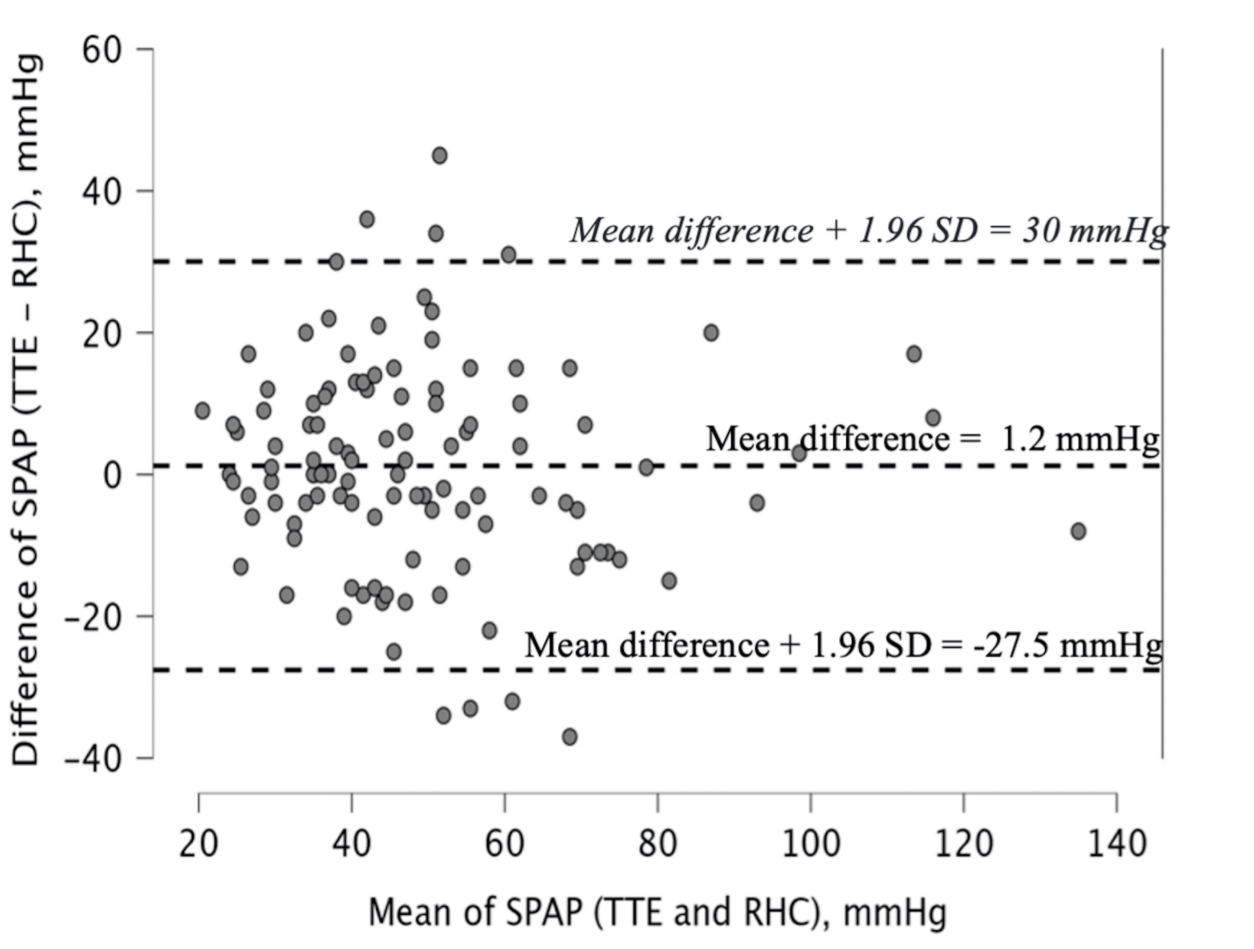
Bland-Altman Analysis of the Agreement Between Systolic Pulmonary Artery Pressure (SPAP) Estimated by Transthoracic Echocardiography (TTE) and Measured by Right Heart Catheterization (RHC) Bland-Altman plot illustrating the agreement between systolic pulmonary artery pressure (SPAP) estimated by transthoracic echocardiography (TTE) and that measured by right heart catheterization (RHC) in all patients. The central dashed line represents the mean bias (mean difference = 1.2 mmHg), while the upper and lower dashed lines indicate the 95% limits of agreement (mean difference ± 1.96 SD = -27.5 mmHg to 30.0 mmHg). The mean bias is minimal, but the limits of agreement are wide, indicating substantial variability between the two measurement methods.

The Bland-Altman analysis (Figure 4) demonstrated a mean bias of +1.23 mmHg between TTE- and RHC-SPAP, with 95% limits of agreement from -27.6 to +30.0 mmHg. Discrepancy analysis showed that 52 (46.4%) patients differed by >10 mmHg, 15 (13.3%) by >20 mmHg, and eight (7.1%) by >30 mmHg. TTE overestimated RHC values in 52%, 57%, and 50% of cases at discrepancy thresholds of >10, >20, and >30 mmHg, respectively.

In the univariable regression analysis, neither TAPSE (p = 0.239) nor vena contracta (p = 0.746) was associated with ΔSPAP. In the multivariable analysis, the predictors in combination remained nonsignificant (p = 0.496) (Table 3).

**Table 3:** Linear Regression Analysis of Tricuspid Annular Plane Systolic Excursion (TAPSE) and Vena Contracta (VC) in Relation to ΔSPAP Linear regression analysis demonstrating the association of TAPSE and vena contracta with ΔSPAP, defined as the difference between systolic pulmonary artery pressure (SPAP) estimated by TTE and that measured by RHC. Both univariable and multivariable linear regression analyses were performed, and results are presented as β coefficients with corresponding p-values. Neither TAPSE nor vena contracta, individually or in combination, was significantly associated with ΔSPAP. The multivariable model including VC and TAPSE was not statistically significant (p = 0.496), with an R² value of 0.018 (adjusted R² = -0.007).

Predictor	β (Unstandardized)	p	95% CI	
			Lower	Upper
VC (mm)	0.379	0.746	-0.836	1.163
TAPSE (mm)	0.163	0.239	-0.257	1.016

Subgroup analyses

Tricuspid Regurgitation

In patients with mild (n = 31), moderate (n = 44), and severe TR (n = 37), no significant differences were observed between the TTE- and RHC-derived SPAP values (p = 0.67, p = 0.78, and p = 0.14, respectively). The correlations were moderate across the groups (ρ = 0.60, ρ = 0.51, and ρ = 0.62). Bland-Altman analysis demonstrated the narrowest limits of agreement in moderate TR (bias -0.6 mmHg; LoA -26 to +27 mmHg), whereas both mild (bias +0.7 mmHg; LoA -32 to +31 mmHg) and severe TR (bias +3.4 mmHg; LoA -25 to +32 mmHg) were associated with greater variability.

Right Ventricular Function

When stratified by RV function, no significant differences were observed between TTE- and RHC-derived SPAP in patients with preserved (TAPSE ≥17 mm; n = 68; p = 0.21) or reduced RV function (TAPSE <17 mm; n = 44; p = 0.87). The correlation was strong in preserved RV function (ρ = 0.78; p < 0.01) and weaker but still significant in reduced RV function (ρ = 0.48; p < 0.01).

PH Subgroup

**Figure 5:**
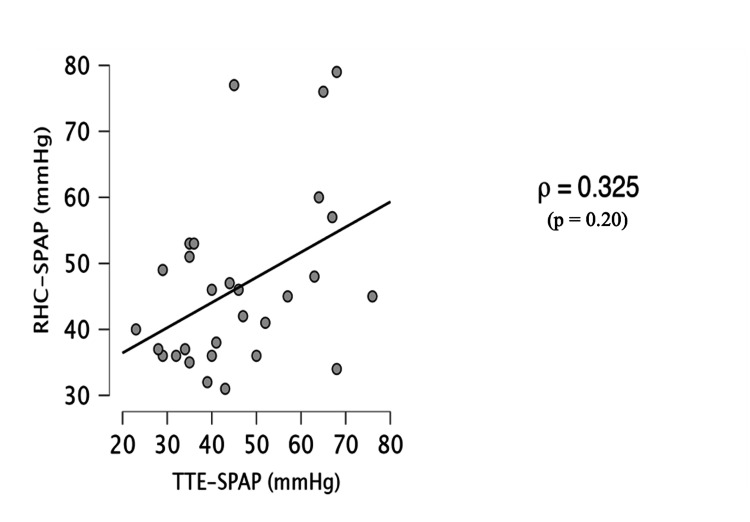
Correlation Between Systolic Pulmonary Artery Pressure (SPAP) Derived From Transthoracic Echocardiography (TTE) and Right Heart Catheterization (RHC) in Patients With Postcapillary Pulmonary Hypertension Scatter plot illustrating the Spearman rank correlation between SPAP estimated by TTE and that measured by RHC in patients with postcapillary pulmonary hypertension. A weak, non-significant correlation was observed (ρ = 0.325, p = 0.20).

When stratified by PH subgroup, the correlation between TTE- and RHC-derived SPAP was significant in precapillary (n = 18; Spearman’s ρ = 0.68; p < 0.01) and combined pre- and postcapillary PH (n = 31; ρ = 0.66; p < 0.001). No significant correlation was observed in postcapillary PH (n = 29; ρ = 0.37; p = 0.20) (Figure 5).

Additional hemodynamic parameters

The noninvasive right atrial pressure and TAPSE/SPAP ratio correlated strongly with invasive measurements (ρ = 0.64 and ρ = 0.78, respectively; both p < 0.001), with no significant differences between the two methods (p = 0.40 and p = 0.14, respectively).

## Discussion

TTE-derived SPAP demonstrated a moderate correlation with invasive RHC-derived SPAP. However, Bland-Altman analysis revealed wide limits of agreement, with nearly half of the patients showing discrepancies >10 mmHg. RV function (TAPSE) and TR severity (VC) did not explain the differences between TTE and RHC. The observed variability is likely multifactorial, and current evidence remains insufficient to fully delineate the determinants of this inaccuracy.

These findings indicate that, while TTE may approximate pulmonary pressures at the population level, it cannot be used interchangeably with RHC for diagnostic or therapeutic decision-making at the individual level.

These findings align with prior studies reporting modest correlations between TTE and RHC SPAP, with significant variability [17-22]. Previous reports have demonstrated that distorted TR envelopes, elevated right atrial pressure, and altered loading conditions compromise Doppler accuracy [12,19,20], and our results confirm these limitations.

Agreement between TTE- and RHC-derived SPAP was greatest in moderate TR, whereas both mild and severe TR were associated with greater variability; in particular, severe TR frequently resulted in overestimation, consistent with prior studies highlighting the limited reliability of Doppler estimates in severe regurgitation [12,14,15]. The weaker correlation in patients with reduced RV function (TAPSE <17 mm) compared with preserved RV function may be related to systolic dysfunction, which introduces complex flow patterns that impair Doppler accuracy and may contribute to wider measurement differences [2,5]. In patients with isolated postcapillary PH, no correlation was observed between TTE- and RHC-derived SPAP, likely reflecting the distinct pathophysiology of this phenotype, in which SPAP elevation results primarily from increased left atrial and pulmonary venous pressures rather than elevated PVR [1,2]. Consequently, higher left-sided pressures may not correspond to increased TR jet velocity [11], leading to Doppler-derived right ventricle-right atrium (RV-RA) gradients underestimating the true SPAP and likely contributing to considerable measurement discrepancies in this subgroup [11,13,19-21]. Moreover, RAP estimates based on IVC dynamics may be disproportionately low relative to pulmonary venous and left atrial pressures in this setting [8,16], and the frequent coexistence of atrial fibrillation can further impair accuracy [1]. Collectively, these mechanisms underscore the particular limitations of echocardiography in postcapillary PH and reinforce the role of invasive hemodynamics for accurate classification and clinical decision-making [1,19,20,22].

Limitations

This study had several limitations. First, its retrospective, single-center design may restrict the generalizability of our findings. Second, because TTE and RHC were not consistently performed on the same day, interval changes in volume status or hemodynamic conditions - including possible effects of intravenous diuresis - may have influenced the observed results. Third, Doppler-derived SPAP estimates are limited by technical challenges, particularly in patients with atrial fibrillation or eccentric TR jets.

Fourth, RV function was assessed solely by TAPSE, whereas more advanced indices, such as RV strain or three-dimensional echocardiography, may provide additional insights. Fifth, TR severity was evaluated only by VC width, which may not fully capture the complexity of regurgitant lesions. Finally, subgroup analyses, especially across PH phenotypes, were limited by sample size and should therefore be interpreted with caution.

Clinical implication

This study highlights the limitations of TTE in estimating SPAP. While TTE remains valuable for PH screening and longitudinal follow-up, reliance on TTE alone may lead to diagnostic inaccuracy, particularly in patients with severe TR, RV dysfunction, or postcapillary PH. These findings reinforce the need for RHC as the gold standard for accurate diagnosis, risk stratification, and therapeutic decision-making in PH.

## Conclusions

TTE provides a useful noninvasive estimate of SPAP but remains limited in terms of accuracy and consistency in specific clinical settings. The lack of predictive value of RV function and TR severity suggests that other physiological factors underlie the observed discordance with RHC. Therefore, right heart catheterization should remain the reference standard for precise assessment of pulmonary artery pressure.
